# Impact of chronic kidney disease and anemia on health-related quality of life and work productivity: analysis of multinational real-world data

**DOI:** 10.1186/s12882-020-01746-4

**Published:** 2020-03-07

**Authors:** Heleen van Haalen, James Jackson, Bruce Spinowitz, Gary Milligan, Rebecca Moon

**Affiliations:** 1 AstraZeneca, Gothenburg, Sweden; 2 Adelphi Real World, Bollington, UK; 3 Division of Nephrology, New York Presbyterian Queens, Flushing, NY USA

**Keywords:** Anemia, Chronic kidney disease, EQ-5D-3 L, Health-related quality of life, KDQOL-36, Patient-reported outcomes, Real-world

## Abstract

**Background:**

Reductions in health-related quality of life (HRQoL) in patients with chronic kidney disease (CKD) are thought to be exacerbated by the low hemoglobin (Hb) levels that define anemia, a common complication of CKD. The current analysis evaluated the impact of anemia on HRQoL and work productivity in patients with non-dialysis dependent and dialysis-dependent CKD using real-world data.

**Methods:**

Data were collected in France, Germany, Italy, Spain, the UK, the USA and China in 2012–2018 in the Adelphi Real World Disease Specific Programme™ for CKD, a large, cross-sectional, survey of physicians and their patients. Patients completed three patient-reported outcomes (PRO) instruments: the EuroQol 5-Dimension 3-level (EQ-5D-3 L), the Kidney Disease Quality of Life (KDQOL-36) instrument and the Work Productivity and Activity Impairment questionnaire. PROs were assessed by CKD stage and Hb levels, and regression analyses were performed with CKD stage and Hb level as independent variables and PROs as outcome variables, while adjusting for age, sex, CKD stage, comorbidities and cardiovascular risk.

**Results:**

Overall, 5276 patients participated in the survey, including 28% stage 4 and 36% dialysis patients. Patients with lower Hb levels more often reported problems/issues on all EQ-5D-3 L domains (*p* < 0.0001). Regression analyses showed significant associations between lower Hb levels and the probability of low (< 0.8) EQ-5D-3 L utility scores (*p* < 0.0001) and low visual analog scale scores (*p* < 0.05), indicating poorer health status. Associations were seen even when adjusting for CKD stage and other potential confounding factors. Significant associations were observed between Hb level and the 12-Item Short-Form Health Survey (SF-12) Physical Component Summary, SF-12 Mental Component Summary and the three KDQOL-36 subscales (all *p* < 0.0001), and were confirmed using linear regression analyses adjusting for CKD stage and other potential confounders. Numerically greater work productivity losses and greater activity impairment were observed with lower Hb levels.

**Conclusions:**

Lower Hb levels worsen the impact of CKD on HRQoL, and are associated with lower work productivity in patients with CKD. Assessment and treatment of anemia should be recognized as a key component of integral CKD management throughout all stages of the disease.

## Background

Chronic kidney disease (CKD) is a progressive condition defined by the presence of kidney damage and decreased level of kidney function, most commonly expressed in terms of estimated glomerular filtration rate (eGFR). CKD has a global prevalence of 11–13% [1], with estimates ranging from 3.9 to 15.3%, depending on geographical region. The lowest estimates have been reported for Europe and the highest for China [2–4]. In 2015, it was estimated that more than 20 million people in the USA had CKD, with the majority having stage 3 disease, and only a small proportion at stage 4 or 5 [5].

Anemia, a decrease in the hemoglobin (Hb) carried within red blood cells, is a common complication of CKD and is associated with debilitating symptoms, including fatigue, weakness, shortness of breath, dizziness, headaches and depression [6, 7]. Anemia has been defined as Hb < 12 g/dL in women and < 13 g/dL in men [8]. Anemia in CKD is predominantly caused by a relative deficiency of erythropoietin, a hormone produced in adults primarily by the kidneys [9], although shortened red blood cell half-life and functional iron deficiency also contribute to the anemia of CKD [10]. In patients with CKD, anemia is also known to be associated with increased cardiac output, development of left ventricular hypertrophy, angina and congestive heart failure, which in turn are associated with increased morbidity and mortality [11–16]. Analysis of data from the US National Health and Nutrition Examination Survey indicated the proportion of CKD patients with anemia, using the Kidney Disease Improving Global Outcomes definition, was 15.4%; double that in the general population (7.6%), increasing from 8.4% at stage 1 to 53.4% at stage 5 [17]. In addition, several other studies have shown that mean Hb levels decline with deteriorating kidney function [8, 18].

Health-related quality of life (HRQoL) is known to be reduced in patients with CKD, and the presence of anemia appears to be associated with exacerbation of HRQoL impairment [19–22]. However, the incidence of anemia increases with deteriorating kidney function, as do other complications of CKD. It can therefore be very difficult to separate the impact of anemia on HRQoL from the impact of other factors using observational data, although data from large patient cohorts with multiple variables can aid understanding of how patients are affected and provide new insights. Moreover, there are few reports of analysis of data from large cohorts of patients across various geographical areas, using a disease-specific instrument to assess HRQoL. The objective of the current survey was to evaluate the impact of anemia on HRQoL in patients with CKD at varying stages of disease using real-world data from different geographical locations.

## Methods

### Survey design and data collection

Data were collected in Europe (France, Germany, Spain, Italy, the UK), the USA and China during three periods between June 2012 and February 2018, using the Adelphi Real World Disease Specific Programme™ (DSP) for CKD. DSPs are large, real-world, cross-sectional, multi-country surveys of physicians and their consulting patients in clinical practice [23]; a diagram describing the study design can be found in Additional file 3: Fig. 1.

Physicians were identified from publicly available lists and invited to participate in the DSP following completion of a short screening questionnaire, if they met the following eligibility criteria:

- Were nephrologists, endocrinologists, cardiologists, hematologists or primary care physicians.
- Were actively involved in managing the treatment of patients with CKD.
- In a typical month, saw a specified minimum number of patients with different stages of CKD (the required number varied between data collection periods and depended on their specialty).
  Participating physicians were asked to complete a Patient Record Form (PRF) for twelve patients with CKD stage 3a or above, with a specified number of patients at each stage of CKD (the numbers required at each stage varied depending on the data collection period and the physician’s specialty). EGFR was used for both diagnosis and staging of CKD, with eGFR ≥90, 60–89, 45–59, 30–44, 15–29 and < 15 ml/min/1.73 m2 representing stages 1, 2, 3a, 3b, 4 and 5, respectively [8]. PRFs were completed for consecutively consulting patients at each stage of CKD until the quota of patients for that stage had been reached. Information recorded in the PRF included demographics; disease characteristics and history; current Hb level; concomitant conditions; current treatment and treatment history. Patients for whom a PRF was completed were also invited to complete a patient self-completion form (PSC), which included complementary information on CKD history to that recorded on the PRF, as well as a number of well-established patient-reported outcomes (PRO) instruments*.*
  Data were collected according to market research guidelines; hence, no source validation was possible or required. Patient and physician identities were not known to the research team; no identifiers were recorded for the patients, and PRFs and PSCs for each patient were linked by unique numeric codes pre-printed on the forms.

### PRO questionnaires

HRQoL was assessed using the generic EuroQol 5-Dimension 3-level (EQ-5D-3 L) measure of health status and the disease-specific Kidney Disease Quality of Life (KDQOL-36) instrument. Productivity was also assessed using the Work Productivity and Activity Impairment (WPAI) questionnaire.

The EQ-5D-3 L is a generic instrument often used routinely in healthcare systems to assess patient health status before and after an intervention [24]. It comprises 5 individual items and a 20 cm vertical visual analog scale (VAS) [25, 26]. The individual items ask the respondent to indicate the level of problems related to mobility, self-care, and usual activities (e.g. work, study, housework, family or leisure activities), and the severity of pain/discomfort and anxiety/depression experienced (if any). Each item provides a score ranging from 1 to 3; a single health utility index score is generated using a country-specific algorithm that provides a number, with 1 indicating perfect health, 0 death and < 0 worse than death [27]. Patients indicate their general health status on the day that they complete the EQ-5D-3 L by drawing a line on the VAS to provide a score ranging from 0 (worst imaginable health state) to 100 (best imaginable health state).

The KDQOL-36 is one of the most-commonly used disease-specific instruments in studies showing HRQoL impairment in CKD [28–31]. It comprises 36 items, including the generic 12-Item Short-Form Health Survey (SF-12) to provide 2 summary scores assessing impact on the physical and mental dimensions of HRQoL, and a further 24 items to provide 3 disease-specific subscales [32, 33]:

- The *SF-12 Physical Component Summary (PCS)* is calculated from all 12 items in the SF-12.
- The *SF-12 Mental Component Summary (MCS)* is calculated from all 12 items in the SF-12.
- The *symptoms and problems with kidney disease subscale* is calculated from 12 items each describing a symptom of kidney disease. Patients are asked to what extent they were bothered by each of these during the past 4 weeks, with 5 response options ranging from “Not at all bothered” to “Extremely bothered.”
- The *effects of kidney disease on daily life subscale* is calculated from 8 items describing ways in which kidney disease can impact a range of issues, such as a patient’s ability to work around the house, and their personal appearance. Patients are asked to what extent they are bothered by each of these, with 5 response options ranging from “Not at all bothered” to “Extremely bothered”.
- The *burden of kidney disease subscale* is calculated from 4 statements related to the impact of kidney disease on the patient and their family. Patients indicate their agreement with the statements by choosing from 5 response options ranging from “Definitely true” to “Definitely false”.
  The SF-12 PCS and MCS are calculated as the sum of scores following conversion into standardized values. The disease-specific subscales are scored by transforming all items to a score in the range 0 to 100 and averaging across the items. Higher scores indicate better HRQoL in all cases.

The WPAI is an instrument to assess the impact of disease on work productivity and daily activities over the past 7 days. Its use has been completed by patients in a large number of studies and a wide range of disease areas [34]; as it is not disease-specific, it can be used to compare productivity impact across diseases. The WPAI comprises 6 items and results in the generation of 4 scores, each expressed as a percentage of work time missed or a percentage impairment [35]:

- Absenteeism (work time missed due to impairment): calculated as hours missed as a percentage of total work hours using: patient-reported hours missed during the 7-day recall period / (patient-reported hours worked during the 7-day recall period + patient-reported hours missed during the 7-day recall period) × 100);
- Presenteeism (ability to function at work while being impaired): calculated as a percentage using: patient-reported impact of CKD on productivity at work during the 7-day recall period recorded on a scale of 0 (no impact) to 10 (prevented me from working) × 10;
- Overall work impairment: calculated as a percentage using: patient-reported hours worked, patient-reported hours missed and patient-reported impact of CKD on productivity during the 7-day recall period, applying an algorithm described on the WPAI website [36]; and
- Total activity impairment: calculated as a percentage using: patient-reported impact of CKD on productivity in regular unpaid activities during the 7-day recall period recorded on a scale of 0 (no impact) to 10 (prevented activities) × 10.

### Analysis

Means and standard deviations were calculated for continuous variables, and frequency counts and percentages for categorical variables. Descriptive analyses were performed for the total survey population and stratified by Hb level, geographical region, and CKD stage. For the generic EQ-5D-3 L, Hb levels of < 8 g/dL, 8- < 10 g/dL, 10–12 g/dL, and > 12 g/dL were used; for the KDQOL-36 and WPAI, Hb levels of < 10 g/dL, 10–12 g/dL, and > 12 g/dL were used. CKD stages were 3a non-dialysis dependent (NDD), 3b NDD, 4 NDD, 5 NDD, and dialysis-dependent (DD).

The non-parametric Spearman’s rank correlation test was used to assess the correlation of Hb level with EQ-5D-3 L utility index and domains, EQ-5D-3 L VAS, SF-12 PCS, SF-12 MCS scores and the three subscales from the KDQOL-36. To adjust for potential confounding, linear regression analyses were performed on EQ-5D-3 L VAS, SF-12 PCS, SF-12 MCS and the three subscales from the KDQOL-36 as the outcome variables; independent variables included were Hb level (continuous), CKD stage, Hb and CKD stage interacted, sex, common comorbidities (diabetes, heart failure, stroke) and cardiovascular risk. Logistic regression analysis was performed with the same independent variables and EQ-5D-3 L utility index score (classified as ≥0.8 and < 0.8) as the outcome variable. Exploratory analyses compared KDQOL-36 and WPAI scores for patients with Hb < 8 g/dL with those for patients with higher Hb levels.

Patients who had completed a PSC, and for whom current CKD stage and Hb level were available were included in the analysis. Patients with no Hb level reported were included in the descriptive analysis of demographics and disease characteristics, but excluded from all other analyses. Missing data were not imputed but remained missing; therefore, the base of patients for analysis could vary between variables, and is reported for each analysis. All descriptive and exploratory analyses were conducted using IBM SPSS Data Collection Survey Reporter version 6 or later and all statistical testing was conducted in Stata v15.1 [37].

## Results

### Participants

Overall, 770 physicians participated, providing data for a total of 5276 patients, 2622 from Europe, 1933 from the USA and 721 from China. Sixty-four percent of patients were NDD and 36% of patients were DD. In addition, 28% of patients were in CKD stage 4, 19% were in CKD stage 3b and 16% were in CKD stage 3a. Only 1% of patients were in CKD stage 5 while NDD. The mean Hb level within the cohort was 11.5 g/dL (standard deviation [SD] 1.9 g/dL) and 13% of patients had a Hb level < 10 g/dL. The range of measured Hb values in the data was 3.4 to 20.5 g/dL. The distribution of Hb values is shown in Additional file 3: Fig. 2. Hypertension was identified as the underlying cause of CKD in 59% of patients, followed by Type 2 Diabetes (39% of patients; Table 1). However, multiple causes of CKD were registered for some patients.

**Table 1:** Patient demographics and disease characteristics

	All patients *N* = 5276	Europe *N* = 2622	USA *N* = 1933	China *N* = 721
Age^a^, years				
Mean (SD)	60.9 (14.3)	62.5 (15.1)	59.8 (14.2)	58.3 (10.1)
Median	62	65	61	59
Min, Max	18, 89	18, 89	18, 89	22, 89
> 90 years old, n (%)	42 (0.8%)	25 (1.0%)	17 (0.9%)	0 (0.0%)
Sex, n (%)				
Female	2259 (43%)	1067 (41%)	869 (45%)	323 (45%)
Male	3017 (57%)	1555 (59%)	1064 (55%)	398 (55%)
BMI, kg/m^2^				
Mean (SD)	26.9 (5.8)	26.5 (4.9)	29.2 (6.4)	22.4 (3.2)
Median	25.9	25.8	28.1	22.0
Min, Max	13, 74	15, 74	13, 70	14, 47
Unknown, n (%)	162 (3.1%)	94 (3.6%)	61 (3.2%)	7 (1.0%)
Ethnic origin, n (%)				
White/Caucasian	2991 (57%)	1928 (74%)	1063 (55%)	0 (0%)
Chinese	754 (14%)	2 (0%)	37 (2%)	715 (100%)
African American	517 (10%)	5 (0%)	512 (27%)	0 (0%)
Hispanic/Latino	248 (5%)	51 (2%)	197 (10%)	0 (0%)
North/Western/Middle EU	437 (8%)	437 (17%)	0 (0%)	0 (0%)
Asian - other	76 (1%)	12 (0%)	64 (3%)	0 (0%)
Other^b^	241 (5%)	181 (7%)	59 (3%)	1 (0%)
Unknown	12 (< 1%)	6 (< 1%)	1 (< 1%)	5 (< 1%)
Employment status, n (%)				
Employed^c^	1265 (24%)	593 (23%)	574 (30%)	98 (14%)
Retired	2555 (49%)	1356 (52%)	719 (37%)	480 (67%)
Other^d^	1287 (24%)	607 (23%)	558 (29%)	122 (17%)
Unknown	169 (3%)	66 (3%)	82 (4%)	21 (3%)
Current Hb level, g/dL				
Mean (SD)	11.5 (1.9)	11.6 (1.8)	11.8 (1.9)	10.8 (1.7)
Median	11.5	11.6	11.5	11.0
Min, Max	3, 21	3, 19	6, 21	4, 19
Unknown, n (%)	485 (9.2%)	214 (8.2%)	247 (12.8%)	24 (3.3%)
Current Hb level, n (%)				
Hb < 8 g/dL	141 (3%)	95 (4%)	17 (1%)	29 (4%)
Hb 8–10 g/dL	533 (10%)	206 (8%)	197 (10%)	130 (18%)
Hb 10–12 g/dL	2464 (47%)	1216 (46%)	834 (43%)	414 (57%)
Hb > 12 g/dL	1653 (31%)	891 (34%)	638 (33%)	124 (17%)
Unknown	485 (9%)	214 (8%)	247 (13%)	24 (3%)
Current CKD stage, n (%)				
Stage 3a NDD	821 (16%)	397 (15%)	274 (14%)	150 (21%)
Stage 3b NDD	983 (19%)	523 (20%)	310 (16%)	150 (21%)
Stage 4 NDD	1492 (28%)	753 (29%)	462 (24%)	277 (38%)
Stage 5 NDD	57 (1%)	31 (1%)	24 (1%)	2 (0%)
DD	1923 (36%)	918 (35%)	863 (45%)	142 (20%)
Underlying cause of CKD^e^, n (%)				
Hypertension	3070 (59%)	1417 (54%)	1285 (67%)	368 (51%)
Type 2 Diabetes	2034 (39%)	928 (36%)	812 (42%)	294 (41%)
Cardiovascular disease	888 (17%)	454 (17%)	359 (19%)	75 (10%)
Glomerulonephritis	883 (17%)	391 (15%)	156 (8%)	336 (47%)
Unknown	34 (1%)	14 (1%)	17 (1%)	3 (0%)
Comorbid conditions^f^, n (%)				
Hypertension	2350 (78%)	1044 (82%)	857 (84%)	449 (62%)
Type 2 diabetes	1199 (40%)	430 (34%)	497 (49%)	272 (38%)
Dyslipidemia	877 (29%)	335 (26%)	449 (44%)	93 (13%)
Coronary heart disease	536 (18%)	206 (16%)	196 (19%)	134 (19%)
Depression	366 (12%)	151 (12%)	175 (17%)	40 (6%)
Anxiety	321 (11%)	152 (12%)	144 (14%)	25 (3%)
Atherosclerosis	319 (11%)	162 (13%)	100 (10%)	57 (8%)
No comorbidities	400 (13%)	131 (10%)	35 (3%)	234 (32%)
Unknown	2260 (43%)	1350 (51%)	910 (47%)	0 (0%)
Type of anemia treatment currently received^g^, n (%)	*N* = 1209	*N* = 548	*N* = 384	*N* = 277
Oral iron only	315 (26%)	77 (14%)	139 (36%)	99 (36%)
IV iron only	79 (7%)	54 (10%)	14 (4%)	11 (4%)
ESA only	258 (21%)	126 (23%)	101 (26%)	31 (11%)
Both oral iron and ESA	227 (19%)	107 (19%)	33 (9%)	87 (31%)
Both IV iron and ESA	330 (27%)	184 (34%)	97 (25%)	49 (18%)

^*a*^*Excluding patients aged ≥ 90 years;*^*b*^*Including Native American, Asian-Indian subcontinent, Middle Eastern, Mixed race, Afro-caribbean, Algerian, African, Maghrebian, Eastern Europe, Southern Europe, Turkey, and ‘other’;*^*c*^*Including full-time and part-time;*^*d*^*Including sick leave, homemaker, student, unemployed, and ‘other’;*^*e*^*Includes only underlying causes reported in ≥ 15% of patients - note patients may have > 1 underlying cause;*^*f*^*Includes only comorbid conditions reported in ≥ 10% of patients - note patients may have > 1 comorbid condition;*^*g*^*Includes patients currently receiving anemia treatment – note, only includes 2015 & 2018 sample due to formulation data not available in 2012 dataset*

*BMI, body mass index; CKD, chronic kidney disease; DD, dialysis-dependent; ESA, erythropoiesis-stimulating agent; Hb, hemoglobin; IV, intravenous; NDD, non-dialysis dependent; SD, standard deviation*

Patient demographics were generally similar across geographic regions, although body mass index (BMI) was numerically higher in patients from the USA and lower in patients from China compared with those from Europe (Table 1). The proportion of patients currently employed was numerically higher in the USA and lower in China compared to patients in Europe, and the proportion of retired patients was numerically higher in China and lower in the USA compared to patients in Europe (Table 1); this might reflect geographical differences in the typical age for retirement. Patients’ ethnic origin can be found in Table 1.

Across countries, there were minor differences in the proportion of patients with low Hb, with the proportion of patients with Hb < 10 g/dL being numerically higher in China compared to the USA and Europe. Glomerulonephritis was reported as the underlying cause of CKD in 47% of Chinese patients compared to 8% of patients in the USA or 15% of patients in Europe; however, Chinese patients appeared to have fewer comorbidities (Table 1).

Different types of anemia treatments currently used by participating patients were recorded across regions. However, only data from 2015 and 2018 on anemia treatment types were available (Table 1).

### Health status assessed with EQ-5D-3 L

An association was observed between EQ-5D-3 L domain scores and Hb levels; all EQ-5D-3 L domains showed significantly greater problems/issues with lower Hb levels (*p* < 0.0001 all domains; Table 2).

**Table 2:** EQ-5D-3 L domain scores by Hb level

N n (%)	All Hb levels	Hb > 12 g/dL	Hb 10–12 g/dL	Hb 8–< 10 g/dL	Hb < 8 g/dL	*p*-value^a^
**Mobility**	4665	1613	2387	525	140	P < 0.0001
I have no problems in walking about	2988 (64.1%)	1177 (73.0%)	1453 (60.9%)	293 (55.8%)	65 (46.4%)	
I have some problems in walking about	1613 (34.6%)	429 (26.6%)	901 (37.7%)	216 (41.1%)	67 (47.9%)	
I am confined to bed	64 (1.4%)	7 (0.4%)	33 (1.4%)	16 (3.0%)	8 (5.7%)	
**Self-care**	4656	1612	2381	524	139	P < 0.0001
I have no problems with self-care	3645 (78.3%)	1362 (84.5%)	1824 (76.6%)	375 (71.6%)	84 (60.4%)	
I have some problems washing or dressing myself	914 (19.6%)	234 (14.5%)	504 (21.2%)	129 (24.6%)	47 (33.8%)	
I am unable to wash or dress myself	97 (2.1%)	16 (1.0%)	53 (2.2%)	20 (3.8%)	8 (5.8%)	
**Usual activities**	4652	1610	2379	524	139	P < 0.0001
I have no problems with performing my usual activities	2544 (54.7%)	1031 (64.0%)	1228 (51.6%)	233 (44.5%)	52 (37.4%)	
I have some problems with performing my usual activities	1910 (41.1%)	543 (33.7%)	1049 (44.1%)	249 (47.5%)	69 (49.6%)	
I am unable to perform my usual activities	198 (4.3%)	36 (2.2%)	102 (4.3%)	42 (8.0%)	18 (12.9%)	
**Pain/Discomfort**	4653	1611	2377	524	141	P < 0.0001
I have no pain or discomfort	1943 (41.8%)	825 (51.2%)	899 (37.8%)	178 (34.0%)	41 (29.1%)	
I have moderate pain or discomfort	2534 (54.5%)	744 (46.2%)	1382 (58.1%)	313 (59.7%)	95 (67.4%)	
I have extreme pain or discomfort	176 (3.8%)	42 (2.6%)	96 (4.0%)	33 (6.3%)	5 (3.5%)	
**Anxiety/Depression**	4650	1611	2373	526	140	P < 0.0001
I am not anxious or depressed	2657 (57.1%)	1002 (62.2%)	1294 (54.5%)	284 (54.0%)	77 (55.0%)	
I am moderately anxious or depressed	1769 (38.0%)	556 (34.5%)	946 (39.9%)	210 (39.9%)	57 (40.7%)	
I am extremely anxious or depressed	224 (4.8%)	53 (3.3%)	133 (5.6%)	32 (6.1%)	6 (4.3%)	

^*a*^*Spearman correlation used to compare findings between patients with different Hb levels for each domain*

*Hb, hemoglobin*

**Fig. 1:**
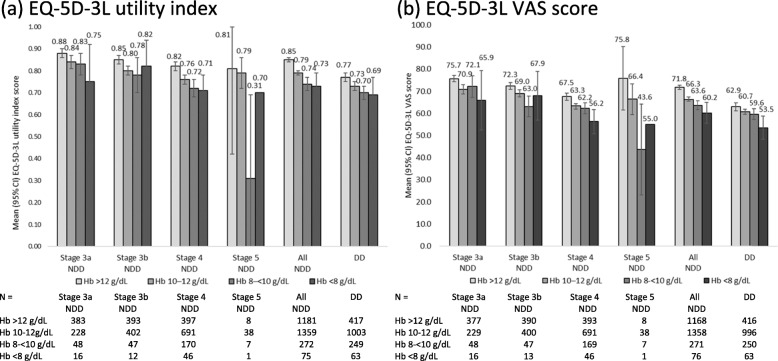
EQ-5D-3 L by Hb level and CKD stage. *CI, confidence interval; CKD, chronic kidney disease; DD, dialysis-dependent; Hb, hemoglobin; NDD, non-dialysis dependent; VAS, visual analog scale*

**Fig. 2:**
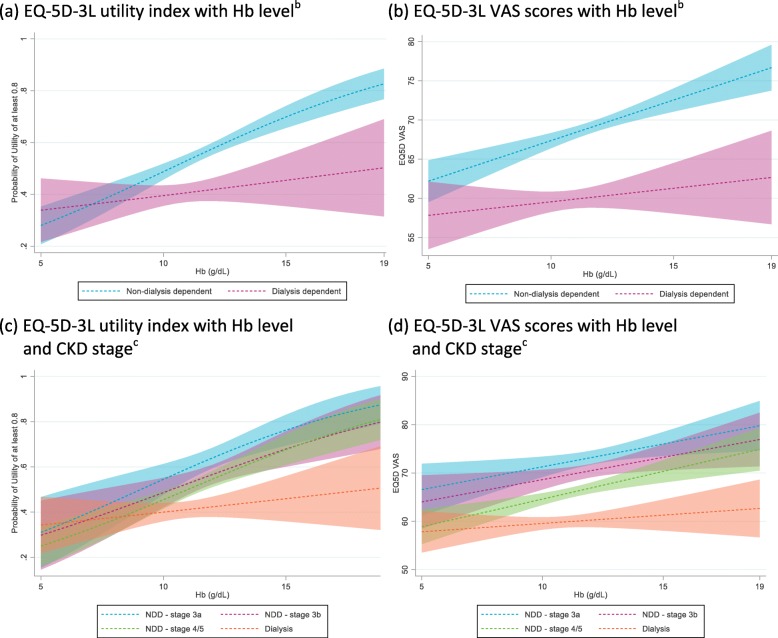
Regression analyses^a^. ^*a*^*These figures use regression models to show how the outcome changes with changing Hb with all other covariates set to their mean values.*^*b*^*Predictive margins with 95% CIs – shown separately for DD and NDD.*^*c*^*Predictive margins of current stage with 95% CIs – shown separately for each CKD stage. CI, confidence interval; CKD, chronic kidney disease; Hb, hemoglobin; NDD, non-dialysis dependent; VAS, visual analog scale*

A trend was observed for lower EQ-5D-3 L utility index and VAS scores, indicating poorer health status, to be reported at more advanced CKD stages and by patients with lower Hb levels across all CKD stages (Fig. 1), although patient numbers at more advanced CKD stage and lower Hb levels were low. Significant associations of Hb level with EQ-5D-3 L utility index score and VAS score were shown using Spearman’s correlations (*p* < 0.0001) and confirmed using logistic regression (EQ-5D-3 L utility index (≥0.8): *p* < 0.0001; Fig. 2a) and linear regression (EQ-5D-3 L VAS: *p* < 0.0001; Fig. 2b). Regression analyses also confirmed associations of EQ-5D-3 L utility index and VAS scores with Hb level and CKD stage (Fig. 2c and d; *p*-values all four analyses < 0.0001). The associations of the HRQoL indices with Hb level were consistently stronger in NDD CKD patients than in DD CKD patients.

The association of lower EQ-5D-3 L utility index and VAS scores with lower Hb levels and more advanced CKD stages was observed across geographic regions (data shown in Appendix – Additional file 1: Table 1). At any Hb level, EQ-5D-3 L utility index scores were generally numerically higher in patients from the USA than those from Europe or China in both NDD and DD patients, while VAS scores were also typically numerically higher in DD patients from the USA than from other geographic regions.

### HRQoL assessed with KDQOL-36

**Fig. 3:**
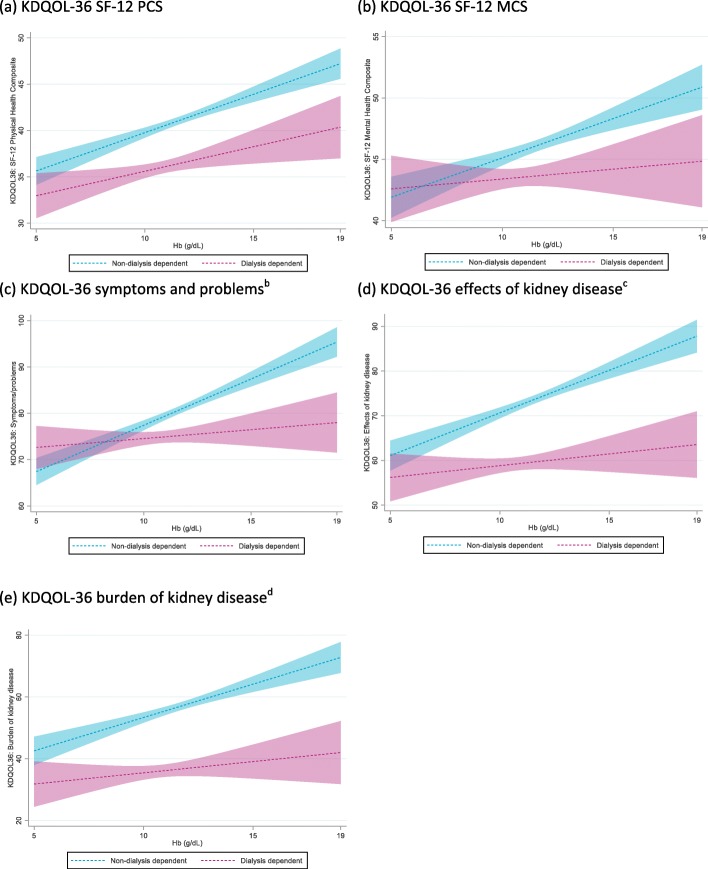
Regression analyses^a^. ^*a*^*These figures use regression models to show how the outcome changes with changing Hb with all other covariates set to their mean values. Predictive margins with 95% CIs are shown separately for DD and NDD*^*b*^*Symptoms and problems with kidney disease subscale.*^*c*^*Effects of kidney disease on daily life subscale.*^*d*^*Burden of kidney disease subscale*. *CI, confidence interval; Hb, hemoglobin; NDD, non-dialysis dependent*

**Fig. 4:**
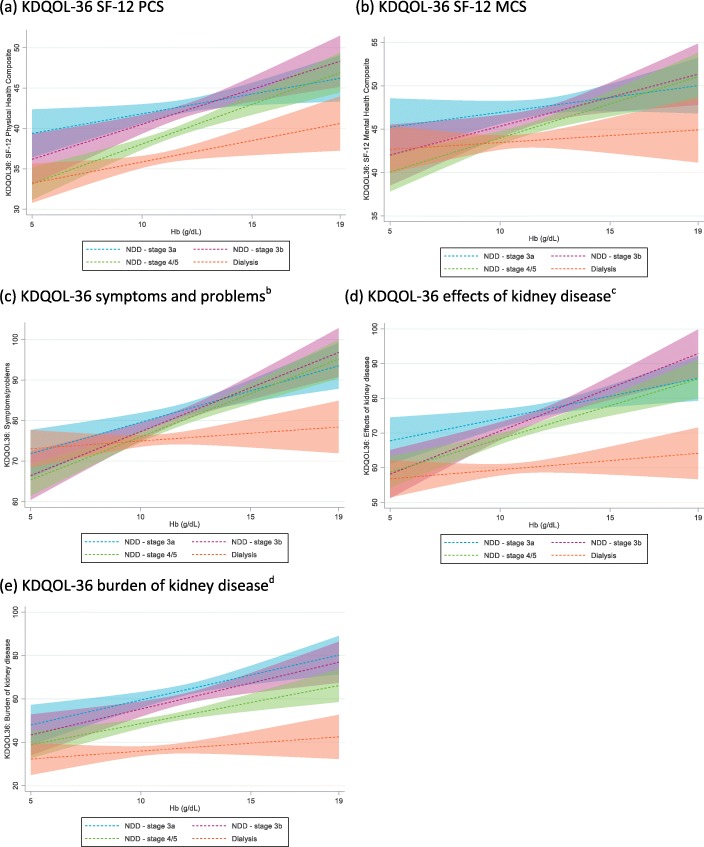
Regression analyses^a^. ^*a*^*These figures use regression models to show how the outcome changes with changing Hb with all other covariates set to their mean values. Predictive margins of current stage with 95% CIs are shown separately for each CKD stage*^*b*^*Symptoms and problems with kidney disease subscale.*^*c*^*Effects of kidney disease on daily life subscale.*^*d*^*Burden of kidney disease subscale*. *CI, confidence interval; Hb, hemoglobin; NDD, non-dialysis dependent*

Numerically lower mean scores, indicating poorer HRQoL, were reported by patients with lower Hb levels for the KDQOL-36 SF-12 PCS, SF-12 MCS, symptoms and problems with kidney disease subscale, effects of kidney disease on daily life subscale, and burden of kidney disease subscale across all CKD stages, with the exception of stage 3a (Table 3). Using Hb level groupings of < 10 g/dL, 10–12 g/dL, and > 12 g/dL, significant associations of Hb level with the SF-12 PCS and MCS scores and all three subscales from the KDQOL-36 were shown using Spearman’s correlations (*p* < 0.0001). These associations were confirmed using linear regression analysis (*p* < 0.0001), with a stronger association observed in NDD than DD patients (Fig. 3a-e). Linear regression analyses also confirmed associations of SF-12 PCS and MCS scores and all three subscales from the KDQOL-36 with CKD stage (p < 0.0001; Fig. 4a–e). Exploratory analyses did not show incremental decreases of KDQOL-36 scores for patients with Hb levels < 8 g/dL compared with those for patients with Hb levels of 8–< 10 g/dL.

**Table 3:** KDQOL-36-3 L scores by Hb level and CKD stage

	N Mean (SD)																							
	Stage 3a NDD				Stage 3b NDD				Stage 4 NDD				Stage 5 NDD				All NDD				All DD			
	All Hb levels	Hb > 12 g/dL	Hb 10–12 g/dL	Hb < 10 g/dL	All Hb levels	Hb > 12 g/dL	Hb 10–12 g/dL	Hb < 10 g/dL	All Hb levels	Hb > 12 g/dL	Hb 10–12 g/dL	Hb < 10 g/dL	All Hb levels	Hb > 12 g/dL	Hb 10–12 g/dL	Hb < 10 g/dL	All Hb levels	Hb > 12 g/dL	Hb 10–12 g/dL	Hb < 10 g/dL	All Hb levels	Hb > 12 g/dL	Hb 10–12 g/dL	Hb < 10 g/dL
**SF-12 PCS**	650 44.7 (9.4)	362 45.8 (9.5)	226 43.3 (9.0)	62 43.0 (9.7)	818 42.8 (9.4)	372 44.6 (9.2)	390 41.6 (9.4)	56 39.7 (9.3)	1271 38.7 (9.5)	384 41.1 (9.7)	680 38.3 (9.2)	207 35.9 (9.2)	48 40.9 (11.1)	7 46.8 (10.6)	34 41.7 (10.6)	7 31.4 (9.5)	2787 41.4 (9.8)	1125 43.8 (9.7)	1330 40.2 (9.5)	332 37.8 (9.7)	1650 37.5 (9.7)	415 38.6 (9.6)	946 37.6 (9.7)	289 35.2 (9.4)
**SF-12 MCS**	650 48.4 (9.4)	362 49.6 (8.9)	226 46.8 (9.6)	62 47.2 (9.8)	818 47.9 (9.3)	372 49.4 (9.0)	390 46.6 (9.3)	56 46.6 (10.5)	1271 45.3 (9.7)	384 47.4 (9.8)	680 44.9 (9.4)	207 43.1 (10.1)	48 45.9 (10.1)	7 49.0 (10.0)	34 47.3 (9.8)	7 35.8 (5.1)	2787 46.8 (9.6)	1125 48.8 (9.3)	1330 45.8 (9.4)	332 44.3 (10.2)	1650 45.5 (10.2)	415 47.3 (9.4)	946 45.2 (10.4)	289 43.7 (10.5)
**Symptoms and problems with kidney disease**	662 84.9 (15.9)	375 87.6 (14.0)	225 81.2 (17.9)	62 82.5 (16.0)	850 82.3 (17.9)	388 86.1 (15.6)	403 79.8 (18.9)	59 74.3 (19.6)	1300 78.5 (17.6)	397 82.5 (16.7)	688 78.1 (16.9)	215 72.6 (19.6)	54 77.1 (20.2)	8 88.8 (13.8)	38 75.7 (20.8)	8 72.4 (20.4)	2866 81.1 (17.6)	1168 85.4 (15.6)	1354 79.0 (17.8)	344 74.7 (19.3)	1747 76.5 (17.2)	420 79.4 (14.9)	1014 76.2 (17.5)	313 73.5 (18.7)
**Effects of kidney disease on daily life**	667 79.1 (18.4)	376 81.9 (17.1)	227 74.9 (19.5)	64 77.7 (19.5)	844 77.6 (19.7)	382 81.5 (17.9)	402 74.4 (20.8)	60 74.1 (18.9)	1300 71.5 (19.6)	397 75.4 (18.9)	689 70.7 (19.5)	214 66.8 (20.4)	54 76.4 (23.6)	8 83.2 (25.7)	38 77.6 (24.5)	8 63.8 (12.5)	2865 75.2 (19.8)	1163 79.6 (18.3)	1356 72.7 (20.1)	346 70.0 (20.3)	1755 63.2 (21.0)	421 67.7 (18.3)	1019 62.2 (21.9)	315 60.4 (20.9)
**Burden of kidney disease**	681 66.4 (26.6)	387 71.1 (24.6)	230 60.0 (27.6)	64 60.8 (29.4)	857 63.3 (27.7)	391 69.1 (25.8)	405 59.3 (28.4)	61 52.9 (27.2)	1319 52.6 (26.6)	401 56.3 (25.5)	702 52.8 (26.4)	216 44.8 (27.5)	54 54.6 (28.6)	8 65.6 (26.3)	38 56.7 (28.5)	8 33.6 (23.1)	2911 59.0 (27.6)	1187 65.4 (26.1)	1375 56.0 (27.4)	349 48.9 (28.4)	1777 40.3 (26.4)	427 40.5 (24.3)	1034 40.8 (27.4)	316 38.3 (25.9)

*CKD, chronic kidney disease; DD, dialysis-dependent; Hb, hemoglobin; MCS, Mental Component Summary; NDD, non-dialysis dependent; PCS, Physical Component Summary; SF-12, 12-Item Short-Form Health Survey; SD, standard deviation*

Mean KDQOL-36 SF-12 PCS and MCS scores were generally numerically higher (indicating better HRQoL) in the USA and lower in China compared to Europe at all stages of CKD, except for SF-12 PCS at stage 5, were patient numbers were very low (data shown in Appendix – Additional file 2: Table 2).

### Productivity assessed with WPAI

Numerically higher mean percentage absenteeism, presenteeism and overall work impairment were reported on the WPAI by patients with lower Hb levels at CKD stages 4 and 5, and for all NDD and DD patients (Table 4). The relatively small number of patients in employment resulted in low samples for calculation of absenteeism, presenteeism and overall work impairment. Increasing levels of total activity impairment were observed with decreasing Hb levels at all CKD stages, except for stage 3a (Table 4). Exploratory analyses indicated greater total activity impairment for patients with Hb levels < 8 g/dL compared with those with Hb levels of 8–< 10 g/dL, but did not show incremental increases in absenteeism, presenteeism, and overall work impairment.

**Table 4:** WPAI scores by Hb level and CKD stage

	N Mean (SD)																							
	Stage 3a NDD				Stage 3b NDD				Stage 4 NDD				Stage 5 NDD				All NDD				All DD			
	All Hb levels	Hb > 12 g/dL	Hb 10–12 g/dL	Hb < 10 g/dL	All Hb levels	Hb > 12 g/dL	Hb 10–12 g/dL	Hb < 10 g/dL	All Hb levels	Hb > 12 g/dL	Hb 10–12 g/dL	Hb < 10 g/dL	All Hb levels	Hb > 12 g/dL	Hb 10–12 g/dL	Hb < 10 g/dL	All Hb levels	Hb > 12 g/dL	Hb 10–12 g/dL	Hb < 10 g/dL	All Hb levels	Hb > 12 g/dL	Hb 10–12 g/dL	Hb < 10 g/dL
**Absenteeism**	210 7.1 (19.9)	141 6.0 (18.6)	57 10.0 (24.0)	12 5.8 (13.1)	167 5.1 (14.8)	98 4.3 (13.5)	61 6.7 (17.3)	8 2.8 (5.4)	239 8.3 (19.9)	104 4.6 (10.3)	103 9.1 (21.8)	32 17.8 (31.4)	9 12.6 (23.0)	3 0.0 (0.0)	5 16.1 (28.5)	1 33.3 (0.0)	625 7.1 (18.7)	346 5.0 (15.0)	226 8.8 (21.4)	53 13.1 (26.0)	290 12.2 (24.0)	106 6.8 (15.5)	147 14.4 (25.9)	37 18.6 (32.7)
**Presenteeism**	234 18.8 (19.8)	164 15.5 17.5	57 25.4 (22.4)	13 30.8 (24.3)	187 19.6 (18.9)	109 18.1 (18.9)	70 22.1 (19.4)	8 17.5 (13.9)	248 27.9 (22.3)	108 25.4 (20.7)	108 26.9 (22.2)	32 39.7 (24.3)	16 21.3 (23.9)	4 7.5 (9.6)	11 22.7 (24.5)	1 60.0 (0.0)	685 22.3 (21.0)	385 18.9 (19.2)	246 25.0 (21.6)	54 34.6 (24.1)	323 34.7 (24.7)	129 31.9 (21.7)	156 35.8 (26.4)	38 39.5 (26.8)
**Overall work impairment**	198 22.1 (22.4)	136 19.5 (20.6)	51 26.9 (24.9)	11 30.6 (27.7)	164 22.5 (21.5)	96 20.3 (21.0)	60 26.4 (22.8)	8 20 (13.4)	231 31.4 (24.7)	102 28.7 (22.8)	100 30.6 (25.0)	29 43.6 (27.2)	9 35.8 (33.8)	3 6.7 (11.6)	5 45.7 (33.2)	1 73.3 (0.0)	602 26.0 (23.6)	337 22.4 (21.7)	216 28.9 (24.6)	49 37.4 (27.0)	283 38.7 (26.4)	106 36.0 (23.8)	144 39.8 (27.5)	33 42.7 (29.3)
**Total activity impairment**	643 30.0 (26.0)	363 25.5 (25.0)	218 36.5 (26.6)	62 33.2 (24.1)	786 34.5 (26.4)	362 28.2 (24.7)	370 39.2 (27.0)	54 44.4 (23.5)	1226 44.4 (26.0)	371 36.3 (24.7)	650 45.4 (25.5)	205 55.9 (25.1)	52 41.2 (30.8)	8 27.5 (28.2)	37 40.3 (30.7)	7 61.4 (27.3)	2707 38.0 (26.9)	1104 30.0 (25.2)	1275 41.9 (26.5)	328 49.8 (26.2)	1621 49.2 (26.5)	398 42.8 (24.5)	936 49.9 (26.9)	287 55.6 (26.0)

*CKD, chronic kidney disease; DD, dialysis-dependent; Hb, hemoglobin; NDD, non-dialysis dependent; SD, standard deviation; WPAI, Work Productivity and Activity Impairment*

## Discussion

This study evaluated the impact of anemia on HRQoL in patients with CKD at varying stages of disease in a large, geographically diverse patient population. CKD itself markedly reduced HRQoL, and this reduction was exacerbated by anemia. At lower Hb levels, similar (low) HRQoL scores were found across CKD stages. This clearly illustrated the humanistic burden of CKD as the disease advanced, particularly for patients with low Hb levels. Using a number of statistical approaches, multiple established HRQoL indices and sub scores showed significant correlations with Hb level. These findings support the idea that Hb level impacts HRQoL in CKD patients, independent of other factors such as CKD stage and other comorbidities, which were included as covariables in the model. Interestingly, the observed correlations between Hb level and HRQoL appeared consistently stronger in NDD CKD patients compared with DD CKD patients. In addition, lower Hb levels were found to be associated with greater work productivity losses and total activity impairments, suggesting potential economic consequences, with increased indirect costs related to the presence of anemia.

Due to the generic nature of the EQ-5D-3 L, comparisons can be made between scores observed for this CKD population and those published for the general population in the same countries. Our findings show that patients with CKD not currently requiring dialysis had EQ-5D-3 L utility index scores similar to the general population (0.80–0.94 [27];) if they had Hb > 12 g/dL, but those with Hb levels indicative of more severe anemia had poorer health status than the general population. For patients requiring dialysis, all utility index scores were lower than population norms, with scores decreasing as Hb level decreased. Compared with population norms for EQ-5D-3 L VAS scores in the relevant countries (72.0–81.6 [27];), VAS scores were lower in this survey for all groups except patients at CKD stages 3a and 3b with Hb > 12 g/dL. The small number of patients with CKD stage 5 limited interpretation of data for this group.

Our results were very consistent, given that lower KDQOL-36 SF-12 PCS scores, SF-12 MCS scores and the scores on the three KDQOL-36 subscales were all significantly associated with lower Hb levels, with regression analyses confirming these associations. The only exception was the lack of an incremental decrement in KDQOL-36 scores at Hb levels < 8 g/dL compared to Hb 10–12 g/dL in exploratory analyses, which might be explained by low patient numbers in this group as well as the limited sensitivity of the KDQOL-36 for common consequences of anemia specifically (fatigue, dizziness, decreased vitality, etc.).

In line with our findings, a number of published studies have reported CKD severity-linked impairment of HRQoL assessed via the EQ-5D-3 L [38, 39] and KDQOL-36 [28, 40]. A number of published studies and a systematic literature review have also reported a correlation of Hb level and HRQoL in patients with CKD [19–21, 29, 41]. However, the correlations reported in these studies are mostly unadjusted for potential confounders and anemia definitions often lack the granularity to differentiate between various levels of severity. Yet these studies, as well as our survey, provide generally consistent evidence on the HRQoL deterioration which occurs with CKD progression and development of anemia.

Various studies have also shown treatment to increase Hb levels in patients with CKD to be associated with HRQoL improvements [42–45]. Improvements were observed in energy/fatigue, physical functioning, ability to work, social activity and cognitive function, although the methods and tools used to measure these changes were diverse across studies, limiting the ability to combine results. Surprisingly, a large, double-blind study of patients with NDD CKD, type 2 diabetes mellitus, and anemia showed only modest improvements in scores from the Functional Assessment of Cancer Therapy–Fatigue instrument, and no improved scores for the 36-Item Short-Form General Health Survey (SF-36) questionnaire, when patients received treatment for their anemia [46]. Additionally, a recent systematic review and meta-analysis concluded that there were no statistically or clinically significant differences between higher and lower Hb targets for HRQoL measured by SF-36 and Kidney Dialysis Questionnaire [47]. However, inherent to its focus on Hb target levels and specific HRQoL measures, potentially relevant studies were not considered due to the inability to combine results within a meta-analysis framework. Another complicating factor in studying HRQoL improvement in relation to Hb target levels are the risks observed in some studies associated with erythropoiesis-stimulating agents. Targeting an Hb level of 13.5 g/dL with these agents has been shown to be associated with an increased risk of adverse events compared with a target of 11.3 g/dL [48]. Limited improvement in HRQoL in some studies may also reflect baseline levels of HRQoL and/or Hb which were not low enough for improvements to be observed. An analysis of the response to treatment for anemia in two phase 2 studies of patients with NDD and DD CKD showed greater improvements in HRQoL in patients with lower baseline Hb levels [49]. Overall, the diversity in methods as well as results across studies still leaves important questions regarding anemia treatment and HRQoL unanswered.

Some limitations of this survey must be noted. As PRFs were completed for the next 12 consecutively consulting patients with CKD, the sample collected was pseudo-random, rather than a truly random sample. However, we did not identify any indications of systematic differences between our survey population and CKD populations that have been described elsewhere, and therefore we do not expect this to impact our findings and conclusions. Like all studies of this nature, the methodology relies on accurate reporting by physicians and patients. Hb levels reported for some patients were very low (two patients had Hb < 4 g/dL and 11 had Hb in the range 4.0 to 4.8 g/dL); these might have resulted from health issues, such as blood loss, unrelated to CKD, but some might have resulted from transcription errors in recording Hb levels in the PRF. It must also be noted that the observational and cross-sectional nature of this survey limits our abilities to assess causality between exposures and outcomes of interest. Therefore, the results from this survey can only be interpreted as associative. Also, patients who had previously received a kidney transplantation were not included in our analysis, thus it was not possible to evaluate the effect of anemia on HRQoL in this subpopulation of CKD patients. Different types of anemia treatment in participating patients were reported across the geographical regions. However, due to the study design no longitudinal comparisons between these were possible. Finally, as this analysis included only patients who had completed a PSC, it is possible that the results might not be generalizable to the broader CKD population, as patients might have been less likely to provide data if they were seriously ill with very poor HRQoL. Whilst acknowledging these limitations, this survey presents one of the largest and richest samples of CKD patients and their HRQoL to date, allowing for a comprehensive description of QoL estimates by Hb level and CKD stage, based on a variety of PRO outcome scales and across geographies. These unique aspects of our survey have advanced our understanding of how both CKD and anemia impact patients’ well-being. Further research exploring HRQoL in patients with CKD receiving treatment to increase Hb levels would provide interesting insights, and could confirm if HRQoL can be maintained or improved through adequate intervention. Given the markedly reduced HRQoL values demonstrated in this survey, such intervention could potentially have very meaningful impact on patients’ daily lives.

## Conclusions

In conclusion, this multinational analysis confirmed a clear and consistent association between the presence of anemia and poorer HRQoL, which was particularly apparent in NDD patients. In addition, a correlation was shown between the presence of anemia and lower productivity, which was observed across all CKD patients irrespective of CKD stage. While aiming for optimal management of patients with CKD, it is clearly important to monitor Hb levels and be aware of the link between anemia and patients’ overall wellbeing.

## Supplementary information

**Additional file 1.** Table 1 EQ-5D-3 L utility index and VAS scores by geographical region, Hb level and CKD stage.

**Additional file 2.** Table 2 KDQOL-36, SF-12 PCS and MCS scores by geographical region, Hb level and CKD stage.

**Additional file 3 **Fig. 1 The three main phases of a Disease Specific Programme (DSP)**.** Figure 2 Distribution of current Hb levels.

## Data Availability

All data supporting the survey is the intellectual property of Adelphi Real World and can be made available upon request (james.jackson@adelphigroup.com). The WPAI questionnaire used in our study can be found here: http://www.reillyassociates.net/WPAI_General.html.

## Abbreviations

BMI: body mass index
CKD: chronic kidney disease
DD: dialysis-dependent
DSP: Disease Specific Programmes™
eGFR: estimated glomerular filtration rate
EQ-5D-3 L: EuroQol 5-Dimension 3-level
Hb: hemoglobin
HRQoL: health-related quality of life
KDQOL-36: Kidney Disease Quality of Life Instrument
MCS: SF-12 Mental Component Summary
NDD: non-dialysis dependent
PCS: SF-12 Physical Component Summary
PRF: patient record forms
PRO: patient-reported outcomes
PSC: patient self-completion
SD: standard deviation
SF 36: 36-Item Short-Form General Health Survey
SF-12: 12-Item Short Form Health Survey
VAS: visual analog scale
WPAI: Work Productivity and Activity Impairment
