# Supplementary material for: Impact of chronic kidney disease and anemia on health-related quality of life and work productivity: analysis of multinational real-world data
Source: BMC Nephrol. 2020 Mar 7;21:88. doi: 10.1186/s12882-020-01746-4 (PMC7060645; doi:10.1186/s12882-020-01746-4)
Supplement: Supplementary file 1 — Additional file 1. Table 1 EQ-5D-3 L utility index and VAS scores by geographical region, Hb level and CKD stage. [file 12882_2020_1746_MOESM1_ESM.docx]

**Additional Table 1** EQ-5D-3L utility index and VAS scores by geographical region, Hb level and CKD stage

|  | **N**  **Mean (SD)** | | | | | | | |
| --- | --- | --- | --- | --- | --- | --- | --- | --- |
|  | **EQ-5D-3L utility index score** | | | | **EQ-5D-3L VAS score** | | | |
|  | **All Hb levels** | **Hb**  **>12 g/dL** | **Hb**  **10–12 g/dL** | **Hb**  **<10 g/dL** | **All Hb levels** | **Hb**  **>12 g/dL** | **Hb**  **10–12 g/dL** | **Hb**  **<10 g/dL** |
| **Europe** |  |  |  |  |  |  |  |  |
| **Stage 3a NDD** | 325 0.87 (0.21) | 194 0.89 (0.18) | 99 0.83 (0.23) | 32 0.81 (0.28) | 324 72.3 (16.7) | 193 74.5 (15.1) | 99 68.5 (17.4) | 32 71.1 (21.5) |
| **Stage 3b NDD** | 456 0.82 (0.26) | 232 0.83 (0.24) | 190 0.80 (0.27) | 34 0.76 (0.30) | 457 68.0 (17.4) | 233 70.0 (16.2) | 190 66.7 (18.1) | 34 62.5 (18.9) |
| **Stage 4 NDD** | 679 0.76 (0.26) | 220 0.81 (0.23) | 366 0.76 (0.24) | 93 0.66 (0.34) | 678 61.4 (17.7) | 220 66.1 (17.1) | 365 60.4 (17.2) | 93 54.5 (17.9) |
| **Stage 5 NDD** | 30 0.72 (0.39) | 6 0.74 (0.54) | 20 0.83 (0.21) | 4 0.17 (0.45) | 30 61.2 (25.0) | 6 70.7 (16.7) | 20 64.1 (24.7) | 4 32.5 (19.4) |
| **All NDD** | 1490 0.80 (0.25) | 652 0.84 (0.22) | 675 0.78 (0.25) | 163 0.70 (0.34) | 1489 65.8 (18.1) | 652 70.0 (16.5) | 674 63.5 (18.0) | 163 58.9 (20.3) |
| **DD** | 829 0.70 (0.31) | 205 0.73 (0.29) | 491 0.69 (0.32) | 133 0.71 (0.29) | 831 56.7 (19.9) | 206 60.2 (19.5) | 491 56.1 (20.1) | 134 53.6 (18.8) |
| **USA** |  |  |  |  |  |  |  |  |
| **Stage 3a NDD** | 212 0.87 (0.14) | 140 0.88 (0.15) | 56 0.87 (0.14) | 16 0.78 (0.11) | 208 76.3 (15.3) | 135 78.2 (14.2) | 57 74.4 (16.7) | 16 67.3 (16.3) |
| **Stage 3b NDD** | 252 0.84 (0.16) | 129 0.87 (0.12) | 111 0.81 (0.19) | 12 0.78 (0.18) | 247 73.3 (15.2) | 125 76.3 (13.40) | 109 71.2 (16.4) | 13 62.5 (14.4) |
| **Stage 4 NDD** | 354  0.79 (0.18) | 139  0.82 (0.16) | 154  0.77 (0.19) | 61  0.79 (0.19) | 350  65.5 (17.5) | 135  68.8 (16.3) | 155  63.6 (18.0) | 60  63.3 (17.7) |
| **Stage 5 NDD** | 22 0.73 (0.22) | 2 1.00 (0.00) | 16 0.74 (0.19) | 4 0.56 (0.30) | 22 69.8 (18.4) | 2 91.0 (8.5) | 16 70.3 (18.0) | 4 57.5 (15.0) |
| **All NDD** | 840  0.82 (0.17) | 410  0.86 (0.15) | 337  0.80 (0.19) | 93  0.78 (0.19) | 827  70.7 (16.9) | 397  74.5 (15.3) | 337  68.2 (17.8) | 93  63.6 (16.8) |
| **DD** | 763 0.78 (0.19) | 207 0.80 (0.17) | 445 0.78 (0.19) | 111 0.74 (0.22) | 754 65.6 (18.4) | 205 65.5 (17.8) | 438 66.5 (18.1) | 111 62.5 (20.4) |
| **China** |  |  |  |  |  |  |  |  |
| **Stage 3a NDD** | 138 0.86 (0.18) | 49 0.87 (0.16) | 73 0.85 (0.20) | 16 0.85 (0.18) | 138 72.2 (13.0) | 49 73.2 (12.2) | 73 71.4 (12.6) | 16 72.8 (17.7) |
| **Stage 3b NDD** | 146 0.80 (0.15) | 32 0.85 (0.14) | 101 0.78 (0.15) | 13 0.86 (0.13) | 146 71.6 (11.9) | 32 74.1 (11.3) | 101 71.0 (12.4) | 13 70.0 (9.6) |
| **Stage 4 NDD** | 271 0.77 (0.20) | 38 0.88 (0.14) | 171 0.76 (0.18) | 62 0.74 (0.27) | 271 69.3 (10.7) | 38 71.7 (13.8) | 171 69.1 (10.2) | 62 68.3 (10.0) |
| **Stage 5 NDD** | 2 0.76 (0.00) | 0  0.00 (0.00) | 2 0.76 (0.00) | 0 0.00 (0.00) | 2 60.0 (0.00) | 0 0.00 (0.00) | 2 60.0 (0.00) | 0 0.00 (0.00) |
| **All NDD** | 557 0.80 (0.19) | 119 0.87 (0.15) | 347 0.78 (0.18) | 91 0.78 (0.24) | 557 70.6 (11.7) | 119 73.0 (12.4) | 347 70.1 (11.4) | 91 69.3 (11.6) |
| **DD** | 140 0.64 (0.27) | 5 0.73 (0.12) | 67 0.65 (0.21) | 68 0.62 (0.32) | 140 59.5 (15.4) | 5 66.8 (4.6) | 67 57.3 (14.3) | 68 61.2 (16.7) |

CKD, chronic kidney disease; DD, dialysis-dependent; Hb, hemoglobin; NDD, non-dialysis; SD, standard deviation; VAS, visual analog scale
