# Supplementary material for: Impact of chronic kidney disease and anemia on health-related quality of life and work productivity: analysis of multinational real-world data
Source: BMC Nephrol. 2020 Mar 7;21:88. doi: 10.1186/s12882-020-01746-4 (PMC7060645; doi:10.1186/s12882-020-01746-4)
Supplement: Supplementary file 2 — Additional file 2. Table 2 KDQOL-36, SF-12 PCS and MCS scores by geographical region, Hb level and CKD stage. [file 12882_2020_1746_MOESM2_ESM.docx]

**Additional Table 2** KDQOL-36, SF-12 PCS and MCS scores by geographical region, Hb level and CKD stage

|  | **N**  **Mean (SD)** | | | | | | | |
| --- | --- | --- | --- | --- | --- | --- | --- | --- |
|  | **SF-12 PCS score** | | | | **SF-12 MCS score** | | | |
|  | **All Hb levels** | **Hb**  **>12 g/dL** | **Hb**  **10–12g/dL** | **Hb**  **<10 g/dL** | **All Hb levels** | **Hb**  **>12 g/dL** | **Hb**  **10–12g/dL** | **Hb**  **<10 g/dL** |
| **Europe** |  |  |  |  |  |  |  |  |
| **Stage 3a NDD** | 308  45.5 (9.6) | 181  46.9 (9.0) | 96  43.6 (9.5) | 31  43.2 (11.5) | 308  47.9 (9.4) | 181  49.0 (9.2) | 96  45.9 (8.9) | 31  47.2 (11.1) |
| **Stage 3b NDD** | 428  43.1 (9.6) | 216  43.8 (9.5) | 181  42.7 (9.7) | 31  40.2 (9.7) | 428  47.8 (9.4) | 216  48.9 (9.0) | 181  46.6 (9.6) | 31  47.5 (10.4) |
| **Stage 4 NDD** | 650  39.0 (9.9) | 211  41.5 (9.9) | 353  38.6 (9.7) | 86  34.9 (9.4) | 650  45.1 (9.7) | 211  46.7 (9.6) | 353  44.5 (9.5) | 86  43.6 (10.8) |
| **Stage 5 NDD** | 26  43.3 (10.3) | 5  45.4 (12.5) | 18  45.8 (7.3) | 3  25.2 (3.0) | 26  44.6 (9.6) | 5  45.5 (9.8) | 18  46.2 (8.9) | 3  32.7 (6.5) |
| **All NDD** | 1412  41.8 (10.1) | 613  43.9 (9.8) | 648  40.7 (9.9) | 151  37.5 (10.5) | 1412  46.5 (9.7) | 613  48.2 (9.3) | 648  45.3 (9.4) | 151  44.9 (10.9) |
| **DD** | 799  37.0 (9.9) | 207  38.2 (10.3) | 468  37.1 (9.8) | 124  34.5 (9.8) | 799  44.0 (10.5) | 207  46.3 (10.2) | 468  43.4 (10.6) | 124  42.6 (10.2) |
| **USA** |  |  |  |  |  |  |  |  |
| **Stage 3a NDD** | 205  45.9 (9.3) | 133  47.3 (8.9) | 57  44.3 (9.6) | 15  39.3 (7.5) | 205  50.5 (9.4) | 133  51.5 (8.7) | 57  49.4 (10.4) | 15  46.3 (10.2) |
| **Stage 3b NDD** | 245  44.2 (9.4) | 125  46.6 (8.7) | 108  42.5 (9.5) | 12  35.3 (8.5) | 245  50.2 (8.7) | 125  50.8 (8.7) | 108  49.6 (8.3) | 12  48.7 (11.8) |
| **Stage 4 NDD** | 350  39.4 (10.4) | 135  41.0 (10.0) | 156  38.5 (10.6) | 59  38.3 (10.4) | 350  47.8 (9.6) | 135  48.6 (9.8) | 156  47.7 (9.5) | 59  46.2 (9.5) |
| **Stage 5 NDD** | 20  38.8 (12.0) | 2  50.3 (3.5) | 14  37.9 (12.7) | 4  36.0 (10.3) | 20  48.7 (10.2) | 2  57.7 (2.2) | 14  50.4 (10.0) | 4  38.1 (2.7) |
| **All NDD** | 820  42.4 (10.3) | 395  44.9 (9.6) | 335  40.7 (10.4) | 90  38.0 (9.7) | 820  49.2 (9.4) | 395  50.3 (9.2) | 335  48.7 (9.3) | 90  46.2 (9.8) |
| **DD** | 714  38.8 (9.6) | 203  39.1 (9.0) | 412  38.9 (9.9) | 99  37.5 (9.4) | 714  48.2 (9.4) | 203  48.4 (8.4) | 412  48.4 (9.4) | 99  47.1 (11.1) |
| **China** |  |  |  |  |  |  |  |  |
| **Stage 3a NDD** | 137  41.0 (8.4) | 48  37.4 (8.4) | 73  42.2 (7.9) | 16  46.2 (6.4) | 137  46.4 (8.6) | 48  46.8 (7.7) | 73  45.9 (9.6) | 16  48.0 (6.7) |
| **Stage 3b NDD** | 145  39.8 (8.2) | 31  42.1 (7.7) | 101  38.7 (8.3) | 13  42.7 (7.8) | 145  44.2 (8.9) | 31  47.3 (9.0) | 101  43.5 (8.8) | 13  42.4 (8.8) |
| **Stage 4 NDD** | 271  37.1 (6.7) | 38  39.2 (7.4) | 171  37.5 (6.3) | 62  35.0 (7.1) | 271  42.7 (9.1) | 38  46.9 (10.4) | 171  43.0 (8.6) | 62  39.5 (8.5) |
| **Stage 5 NDD** | 2  31.6 (2.8) | 0  0.0 (0.0) | 2  31.6 (2.8) | 0  0.0 (0.0) | 2  34.5 (1.7) | 0  0.0 (0.0) | 2  34.5 (1.7) | 0  0.0 (0.0) |
| **All NDD** | 555  38.8 (7.7) | 117  39.2 (8.0) | 347  38.8 (7.5) | 91  38.1 (8.4) | 555  44.0 (9.1) | 117  47.0 (8.9) | 347  43.7 (8.9) | 91  41.4 (8.8) |
| **DD** | 137  33.3 (6.9) | 5  36.3 (7.2) | 66  33.3 (6.1) | 66  33.0 (7.7) | 137  40.0 (8.4) | 5  45.5 (11.3) | 66  38.6 (7.7) | 66  41.0 (8.7) |

CKD, chronic kidney disease; DD, dialysis-dependent; Hb, hemoglobin; MCS, Mental Component Summary; NDD, non-dialysis dependent; PCS, Physical Component Summary; SD, standard deviation; SF-12, 12-Item Short-Form Health Survey
