# Supplementary material for: Impact of chronic kidney disease and anemia on health-related quality of life and work productivity: analysis of multinational real-world data
Source: BMC Nephrol. 2020 Mar 7;21:88. doi: 10.1186/s12882-020-01746-4 (PMC7060645; doi:10.1186/s12882-020-01746-4)
Supplement: Supplementary file 3 — Additional file 3 Fig. 1 The three main phases of a Disease Specific Programme (DSP). Figure 2 Distribution of current Hb levels. [file 12882_2020_1746_MOESM3_ESM.docx]

**Additional Figure 1** The three main phases of a Disease Specific Programme (DSP)

**Additional Figure 2** Distribution of current Hb levels


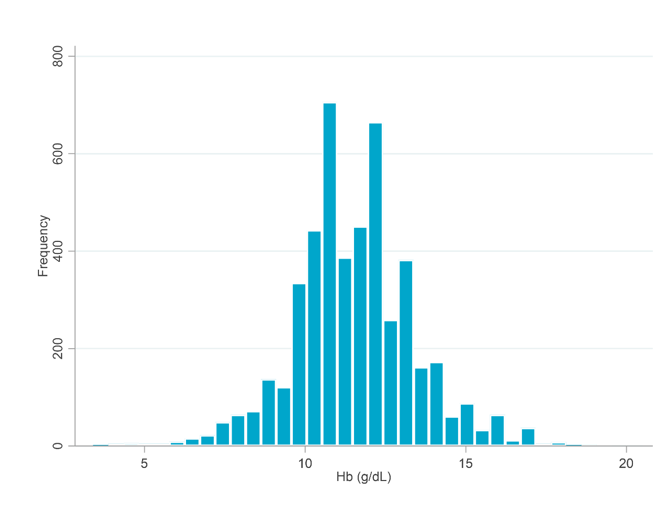


*Hb, hemoglobin*
